# Transparent reporting of research results in *eLife*

**DOI:** 10.7554/eLife.21070

**Published:** 2016-09-09

**Authors:** M Dawn Teare

**Affiliations:** Sheffield School of Health and Related Research, University of Sheffield, Sheffield, United Kingdomm.d.teare@sheffield.ac.uk

**Keywords:** statistical design, reproducibilty, experimental design, scientific publishing, peer review, transparency

## Abstract

Manuscripts should include all the experimental and statistical details that are needed to replicate the experiments and analyses reported in them.

Growing concerns about a lack of reproducibility in certain areas of biomedical research have led to several initiatives to improve the design of experiments, the analysis of data, and the reporting of methods and results (Ioannidis, 2014). Two popular approaches to improving the reliability of published research results have been the pre-registration of experimental protocols and analysis plans, and the introduction of transparent-reporting forms by journals. Such forms are the focus of this editorial.

Pre-registration means that experimental protocols and analysis plans, including blinding and randomisation procedures, are published before any experiments are performed. This is done to reduce bias, to prevent inappropriate post hoc statistical analysis, and to facilitate replication (Chambers and Munafo, 2013; Nosek et al., 2015). The pre-registration approach has evolved to work well in randomised clinical trials and it provides an essential foundation for the systematic reviews that drive evidence-based medicine.

Although a workable framework for pre-registration has yet to emerge for basic science and preclinical studies, various journals have already introduced procedures and checklists to ensure that submitted manuscripts contain all the information an editor, reviewer or reader needs in order to assess the reliability of the results or repeat the experiments (see, for example, Nature, 2013; McNutt, 2014; van *Noorden,* 2014). These journal-specific reporting forms are to be used in conjunction with established reporting guidelines that cover specific types of studies (such as randomised trials, observational studies, systematic reviews and so on: see Equator Network, 2016). This editorial describes the four elements in the transparent reporting form that was introduced by *eLife* last August; authors are required to complete this form before their manuscript is sent for peer review.

**Sample size estimation**: One of the biggest challenges encountered when planning an experiment is to estimate the number of measurements that are required to ensure that the experiment stands a good chance of giving a definitive answer to the question it was designed to address. This number, which is known as the sample size, depends on a number of different factors, including the size of the effect that the researcher expects to see. The lack of any justification of the sample sizes used in experiments is a serious problem in many fields of science, and is a common weakness that has been picked up in a number of recent systematic reviews (Henderson et al., 2015). Estimating the effect size is perhaps the most challenging part of estimating the sample size needed for the experiment (Masca et al., 2015). Further guidance on how to estimate required sample sizes is available in a number of places (see Box 1: Further Resources). It is also important for researchers to take into account the fact that some measurements and/or replicates will fail and, therefore, to increase the initial sample size to counter this.

Box 1. **Further resources**

Guidance on how to estimate sample sizes is available from a number of organizations:

***Equator Network***. http://www.equator-network.org

***Medical Research Council: Cognition and Brain Sciences Unit***. http://imaging.mrc-cbu.cam.ac.uk/statswiki/FAQ/effectSize

***National Centre for the Replacement, Refinement and Reduction of Animals in Research (NC3Rs)***. https://www.nc3rs.org.uk/experimental-design

***National Institutes of Health***. https://www.nih.gov/research-training/rigor-reproducibility/principles-guidelines-reporting-preclinical-research

The *eLife* transparent reporting form is available in both Word and pdf formats.

The eLife transparent reporting form asks authors to state where information about sample sizes (which should include details of the methods used to estimate them and the assumptions made) can be found in their manuscript, or to explain why this information does not apply to their submission.

**Replicates**. The structure of the experiment in terms of how the individual measurements are processed and transformed (including replicated stages) should be presented clearly as a pipeline so that other researchers can replicate the full experiment and understand the statistical analysis. This should include clear rules for the exclusion of samples and the identification of outliers.

**Statistical reporting**. Sufficient details need to be provided within the manuscript for full transparency and replication. The number of measurements and the unit of analysis should be clear for each statistical hypothesis test. The informative display of raw data is also encouraged. When sample sizes are small (N<20 per group), raw data should be displayed graphically rather than as summary statistics. And wherever possible, estimated effect sizes (for example, the difference between two means) should be reported along with 95% confidence intervals, in addition to p-values.

**Additional data files** ("**source data**"). This completes the process of transparency. Raw data and the basic statistical processing scripts used to analyze them can be made available in a number of ways (for example, via the paper itself, GitHub or the Centre for Open Science).

By thinking more carefully and thoroughly about issues like sample sizes, replicates and statistical analysis, by reporting the results of these considerations more fully, and by making data and code available, researchers will increase the confidence of other researchers and the wider world in the robustness and reliability of their published work.
